# Neurodevelopmental and Mental Health Outcomes in Patients With Fontan Circulation: A State-of-the-Art Review

**DOI:** 10.3389/fped.2022.826349

**Published:** 2022-03-09

**Authors:** Johanna Calderon, Jane W. Newburger, Caitlin K. Rollins

**Affiliations:** ^1^PhyMedExp, Université de Montpellier, National Institute of Health and Medical Research (INSERM), CNRS, Montpellier, France; ^2^Department of Psychiatry, Harvard Medical School, Boston, MA, United States; ^3^Department of Cardiology, Boston Children's Hospital, Boston, MA, United States; ^4^Department of Pediatrics, Harvard Medical School, Boston, MA, United States; ^5^Department of Neurology, Boston Children's Hospital, Boston, MA, United States; ^6^Department of Neurology, Harvard Medical School, Boston, MA, United States

**Keywords:** Fontan procedure, neurodevelopment, mental health, brain injury, congenital heart disease (CHD)

## Abstract

Children, adolescents and adults living with Fontan circulation face numerous neurological and developmental challenges. As the population with complex CHD increases thanks to outstanding improvement in medical and surgical care, the long-term developmental and mental health sequelae have become a public health priority in pediatric and congenital cardiology. Many patients with a Fontan circulation experience difficulty in areas of cognition related to attention and executive functioning, visual spatial reasoning and psychosocial development. They are also at high risk for mental health morbidities, particularly anxiety disorders and depression. Several hemodynamic risk factors, beginning during the fetal period, may influence outcomes and yield to abnormal brain growth and development. Brain injury such as white matter lesions, stroke or hemorrhage can occur before, during, or after surgery. Other sociodemographic and surgical risk factors such as multiple catheterizations and surgeries and prolonged hospital stay play a detrimental role in patients' neurodevelopmental prognosis. Prevention and intervention to optimize long-term outcomes are critical in the care of this vulnerable population with complex CHD.

## Introduction

Remarkable advances in pediatric cardiac surgical and medical care in the last few decades have substantially improved survival rates of patients with the most complex forms of congenital heart disease (CHD). Along with excellent survival and improved short-term outcomes has come recognition of long-term neurodevelopmental and psychosocial morbidities (1–4). Individuals with complex CHD, including those with a Fontan circulation, have an elevated risk for structural brain abnormalities and neuropsychological and mental health disorders, starting early in life and continuing throughout adulthood (5, 6).

The etiologies of neurological vulnerability and injury are multifactorial, additive, and interactive. For complex CHD, such as single ventricle anatomies, genetic factors influencing heart formation may also affect brain development. Indeed, whole exome sequencing of CHD parent-offspring trios revealed substantial overlap between damaging de novo mutations in children with CHD and those previously known to be associated with neurodevelopmental disorders (7). In addition, disturbed fetal cerebral hemodynamics may reduce cerebral oxygen and substrate delivery to the developing brain, thereby altering typical brain growth and maturation (8). Patient-specific factors such as lower socioeconomic status, (9) preterm and early-term birth (birth between 37 and 38 weeks' gestation) (10, 11), low birth weight (12), and prolonged hospital stay may further increase risk (13–15).

Compared with other forms of CHD, children with single ventricle disease are reported to be the most vulnerable to neurological sequalae (5, 16). *In utero*, these children often have the lowest cerebral oxygen and nutrient delivery, and after birth, they generally undergo multiple infant and early childhood cardiac operations and catheterizations, potentially exposing the brain to repeated stress due to hemodynamic instability, anesthetic exposure, or other medical complications. These risks persist well beyond the infant/toddler period, as children and adolescents with single ventricle face life-long risk for cerebrovascular events and other systemic morbidities that may impact neurological functioning, mental health, and quality of life.

## Brain Abnormalities For Patients With Single Ventricle CHD

Neuroimaging findings in single ventricle patients across the lifespan range from subtle disturbances in brain maturation and growth to overt injuries evident on clinical imaging. Reductions in brain volumes and other quantitative brain differences emerge as early as the 2nd trimester of pregnancy and persist into adolescence. (17–20) These broad-based developmental disturbances provide a backdrop upon which overt brain injury, such as cerebral white matter injury, acute ischemic stroke, or micro hemorrhage may accumulate over time.

The earliest imaging abnormalities in single ventricle patients emerge in the 2nd trimester of pregnancy. Normal fetal circulation preferentially directs relatively oxygen and nutrient-rich blood to the developing brain while relatively substrate-depleted blood recirculates to the body. In fetuses with single ventricle heart disease, abnormal cardiac anatomy leads to intracardiac mixing, thereby substantially lowering the oxygen and nutrient content of blood directed to the brain. Moreover, in fetuses with hypo plastic or atretic systemic outflow tracts, blood must reach the brain retrograde via the ductus arteriosus. In general, fetuses with CHD show small brain volumes and dysmature gyrification (8, 21–24). In one study, single ventricle heart disease specifically was associated with heightened risk of small total fetal brain volume compared with biventricular forms of CHD (17). Fetal brain size correlates with cerebral oxygen consumption, and regions of brain most vulnerable to low substrate delivery show greatest volumetric reductions (17, 23). These findings lend support to the hypothesis that impaired fetal cerebral oxygen and nutrient delivery plays an important role in these disturbances of brain maturation and growth.

As infants grow into childhood and adolescence, neuroimaging markers of impaired brain growth and development persist. Two large studies of children who underwent the Fontan procedure demonstrate persistent quantitative differences in brain metrics. Watson and colleagues (18) examined 128 children and adolescents who had undergone the Fontan procedure in early childhood. Widespread differences in both regional brain volumes and cortical thickness were present and associated with several medical risks including older age at first operation, more catheterization procedures, and more complications with catheterizations and with surgeries. Separately, the same group assessed an overlapping Fontan cohort for differences in white matter microstructure, again finding widespread differences in white matter microstructure compared to a control group. Of note, these differences in white matter microstructure correlated with Full-Scale IQ and processing speed in the CHD group but not in the control group, suggesting that white matter abnormalities contribute to long-term neurocognitive variability in youth with Fontan circulation (25).

In addition to subtle quantitative differences, clinical brain MRI may detect overt brain injuries. Indeed, one recent study of adolescents and adults with Fontan circulation identified structural brain injury in all 100 participants who underwent brain MRI with the most common injuries being micro hemorrhages (94%) and white matter injury (81%) followed by cerebral infarct (35%), and subcortical gray matter injury (21%) (26).

White matter injury is the most common clinically significant acquired brain injury found in children with single ventricle. About 15–40% of children with single ventricle heart disease have white matter injury visible on MRI prior to surgery, with cumulative injury rates rising as high as 70–80% postoperatively in some studies (20, 26–31). Various factors may contribute to white matter injury, including relative brain dysmaturity (19, 20, 27, 29, 32) and reduced oxygen and nutrient delivery to the developing white matter (27, 33, 34). Perioperative factors including pre- and post-operative hypoxia, longer time to surgery, presence of aortic arch reconstruction, duration of hypothermic cardiac arrest, and postoperative diastolic hypotension have each been shown to correlate with risk of white matter injury. (29, 31, 35, 36) Interestingly, while comparisons are often made between preterm brain injury and white matter injury in CHD, the topology of white matter injury differs between CHD and preterm infants, with CHD infants showing less central injury than preterms (37).

Acute ischemic stroke is the second most common clinically significant brain injury noted in children with single ventricle. Stroke occurs in about 2–20% of children with CHD (29, 38–47). As many children with stroke have no clinical symptoms, higher rates are found in studies where all subjects are screened with MRI compared with studies relying on clinically acquired scans (43, 44, 47). Among patients with CHD, the highest prevalence of stroke is in children with single ventricle; one preoperative study of neonates with HLHS found stroke in 7%, with the rate as high as 13% in a separate study of adolescents who were post-Fontan (27, 48). About a quarter of strokes in children with CHD are associated with catheterization or other cardiac procedures (40, 46, 49, 50). A multitude of factors likely contribute to thromboembolic risk during the periprocedural period including exposure to non-native materials, high hematocrit, inflammation, sluggish venous flow from high Glenn or Fontan pressures, and prolonged immobilization (42, 51). Abnormal heart structure itself and staged palliation create stroke risk due to direct connections between the venous and arterial circulation. For example, at birth, a patent ductus arteriosus, foramen ovale or atrial septal defect allow passage of thrombi from the venous to arterial circulation; similarly, following the stage 1 Norwood procedure and bidirectional Glenn, venous and arterial blood mix in the single ventricle. After Fontan, residual right-to-left shunting may allow venous thrombotic material to pass directly into the arterial circulation. Cumulatively, these risk factors present an important long-term risk for stroke-related morbidity and mortality (39, 52).

Other types of brain injury may also occur in children with single ventricle. Global hypoxic ischemic brain injury is fortunately rare, but may occur in settings of severe hemodynamic compromise. Clinically significant cerebral hemorrhage is also uncommon, but micro hemorrhages are often seen after cardiopulmonary bypass. One study of a biventricular cohort found that a high burden of micro hemorrhage was associated with poorer neurodevelopmental outcome (53). Further study of the clinical implications of this common finding is warranted. Ultimately, patients with single ventricle heart disease face accumulating risk of developmental brain disturbance or injury, with increasing evidence showing associations between these findings and neurodevelopmental outcome (6, 25, 54).

## Neuropsychological and Behavioral Outcomes Throughout the Lifespan

### Outcomes in Children

Children with single ventricle are reported to have the highest risk of neurodevelopmental disability compared with other forms of CHD. More than two decades ago, a study reported outcomes on 11 survivors of HLHS and found that seven children (64%) presented with profound developmental disabilities (55). Wernovsky et al. measured ability and achievement in a cohort of selected survivors whose Fontan procedure was performed in the 1970s and 1980s (55). Children were a median of 11 years of age at assessment and 6 years after surgery. Median Full-Scale IQ was 95.7 ± 17.4, significantly lower than that in the normal population; 8% of patients scored in the severe intellectual dysfunction range (<70), which is about three times the expected proportion in the general population. In multivariable analyses adjusting for social class, lower IQ was primarily associated with the use of circulatory arrest and with the anatomic diagnosis of HLHS or “other” complex forms of single ventricle. Independent risk factors for worse achievement included the diagnosis of HLHS and “Other” complex single ventricle, or prior use of total circulatory arrest, as well as with reoperation using cardiopulmonary bypass within 30 days after the Fontan procedure. In another study of Fontan survivors, Goldberg et al. found a Full-Scale IQ score of 101.4 ± 5.4 within normal ranges for the Fontan group. (56) However, those with HLHS scored significantly lower (93.8 ± 7.3) than those without HLHS, and additional risk factors for worse neurodevelopmental outcome included lower socioeconomic status, longer duration of circulatory arrest, and occurrence of perioperative seizures. In a more contemporary study in 2012, a nationwide sample of 23 patients with HLHS and other univentricular hearts was reported to have a median cognitive performance within the normal range, with only 26 of patients with HLHS and 23% of those with other types of single ventricle having major neurodevelopmental dysfunction (57).

Some studies have reported that, even if at high risk for neurodevelopmental impairments, today, the overall prognosis for patients with single ventricle CHD may not be significantly different from that of other forms of critical CHD. Indeed, it has been suggested that, despite variations in the severity of some symptoms, children with critical CHD share a relative similar neuropsychological and behavioral phenotype. Indeed, a prospective longitudinal study evaluating neurodevelopmental outcomes in 365 young children with CHD at 4 years of age did not find significant differences in unadjusted scores for full-scale IQ (Fontan 93.3 ± 17.1 vs. 96.2 ± 19.8) between survivors of the Fontan procedure and children with CHD who underwent biventricular repair. Scores were also similar for visual perceptive skills, social skills and academic achievement, including math and pre-reading skills. However, preschool children after the Fontan procedure scored significantly lower than those with biventricular repair on processing speed (Fontan 90.8 ± 15.2 vs. 96.5 ± 16.9 for biventricular CHD) and displayed more inattention and impulsive behaviors, as rated by their parents (58).

The Single Ventricle Reconstruction Trial, conducted through the Pediatric Heart Network, was designed primarily to compare outcomes of children with HLHS randomized to a right ventricle to pulmonary artery shunt to outcomes of participants randomized to a Blalock-Thomas-Taussig shunt (59). To date no differences have been detected in neurodevelopmental outcomes between the two shunt groups. Children in this cohort have lower scores on developmental assessments at 14 months and at three years of age. (5, 14) Interestingly, patient specific factors and measures of perioperative morbidities, but not treatment strategies, were found to be predictive of lower developmental scores. (5, 13, 14) Long-term follow-up of the cohort enrolled in the Single Ventricle Reconstruction Trial will likely provide further insights on factors associated with impaired neurodevelopmental outcomes for this high-risk patient group.

### Outcomes in Adolescents

Interestingly, adolescents with a Fontan circulation appear to be at high risk for general cognitive dysfunction and, particularly, for more prevalent behavioral difficulties. Academic achievement (math and reading scores) is also lower than expected in adolescents, suggesting long-lasting learning challenges. A single-center cross-sectional study examined neuropsychological outcomes of a sample of 156 adolescents with Fontan circulation at a mean age of 14 years. Full-scale IQ scores (91.6 ± 16.8), mean Mathematics Composite Score (91.9 ± 17.2) and mean Reading Composite Score (92.0 ± 22.9) were significantly lower than the expected population mean of 100 ± 15. More than a third of the Fontan group scored >1 SD below population means for IQ and both academic achievement tests, and between 12 and 19% scored below−2SD below the norms in these tests. Neurocognitive areas of particular vulnerability in the Fontan group are perceptual reasoning, processing speed and executive function. Memory skills can also be problematic for a proportion of these adolescents. In the Boston adolescent Fontan cohort, scores on the General Memory Index of the Children's Memory Scale were 1 and 2SD below the expected population mean in 34 and 18% of patients respectively, which is higher than expected. Similarly, scores on the General Memory Index of the Wechsler Memory Composite were 1 and 2SD lower in 39 and 14% of patients respectively. Visual spatial skills were lower than the referent group when evaluated with the Rey–Osterrieth Complex Figure, copy and recall trials. Patients who underwent the Norwood procedure scored significantly lower than the non-Norwood group for all scores of the Rey Figure (47).

One of the most vulnerable aspects of cognitive development in individuals with critical CHD including those with a Fontan circulation pertains to executive functioning. Executive functions are a set of higher-order neurocognitive skills that include self-regulation, working memory, behavioral and mental flexibility as well as planning and organization skills. Impairments in executive functioning are common in children and adolescents with CHD, and seem more pronounced for adolescents who underwent the Fontan procedure (60–63). In a study of 463 adolescents, of whom 145 had a Fontan circulation, one-third of parents and teachers scored their behavior in the at-risk range for executive dysfunction on the Behavior Rating Inventory of Executive Function (63). In this study, neuropsychological assessment revealed some variations in the profile for executive functioning across the group with Fontan circulation and those with d-transposition of the great arteries and tetralogy of Fallot. All groups with critical CHD presented with deficits in flexibility/and problem solving; however, scores on visuo-spatial executive function tasks were more altered in the Fontan and TOF groups. Executive function issues are at the core of attention deficit hyperactivity disorder (ADHD) and concern more than a third of adolescents with univentricular hearts. Indeed, in a study of 156 adolescents with Fontan circulation, 38% of them had received a lifetime diagnosis of a disruptive behavior disorder (34 ADHD, 10 oppositional defiant disorder, and 1% adjustment disorder with disturbance of conduct). The proportion with ADHD was significantly higher than that of a same-age referent group without CHD and no differences were found between Fontan adolescents with or without a genetic abnormality (64). In another study of 133 adolescents with Fontan circulation born at term (early, 37–38 weeks' gestation or full-term after 39 weeks' gestation), more than one third of parents reported clinically concerning executive function deficits for Fontan adolescents in their daily lives. This was more prominent for metacognition skills, one of the most complex executive skills, requiring anticipation, organization and planning of one's actions (10). In this study, one third of parents also rated adolescents' ADHD symptoms as clinically concerning, whereas only 7% of the adolescents themselves rated their attention and hyperactivity symptoms as concerning. This discrepancy between parent- and self-reports has been commonly reported in previous studies, (65) suggesting that children and adolescents with critical CHD, including Fontan patients, may struggle with identifying or recognizing their own difficulties.

Importantly, the abnormal developmental milestones observed for executive function skills in childhood and adolescence may predispose the individual with CHD to clinical difficulties in self-adjustment, adherence to treatments and overall lower quality of life (66). Given the importance of deficits in executive function and its relevance in predicting long-term social and health outcomes in youth (65, 67), strategies have been designed to palliate these deficits in the population with CHD (68). These interventions, although with modest overall efficacy, provide hope that, at least some of the issues of self-regulation and working memory may be amenable to intervention in adolescents with critical CHD, including those with Fontan circulation. More research is needed to understand the extent of neurobehavioral plasticity in this at-high risk group.

Finally, social cognition is another neurocognitive area of concern for individuals with critical CHD, including those with a Fontan circulation. Adolescents with d-TGA, tetralogy of Fallot and Fontan circulation have worse scores on tests that assess their ability to interpret the emotions of other people, read social cues and recognize their own emotions (65). Bellinger et al. (47) showed that, compared to a referent group, adolescents after the Fontan procedure had lower scores at the Reading the Mind in the Eyes Test, an assessment of a person's ability to identify the emotions of others from their facial expressions. They also had difficulties identifying their feelings as reported in the self-questionnaire of the Toronto Alexithymia Scale and endorsed more autistic tendencies (i.e., autistic traits) in the Autism Spectrum Quotient self-report. A higher number of catheterizations and older age at developmental assessment were associated with worse outcomes at the Reading the Mind in the Eyes test. Autism traits were significantly associated with the presence of a genetic abnormality and higher number of cardiac surgeries.

Overall, risk factors for adverse neurodevelopmental and behavioral outcomes are highly correlated. They can also be additive and/or cumulative, especially for individuals with single ventricle who present with a chronic health condition. Independent risk factors that have emerged across multiple studies include the presence of genetic disorders, lower birth weight and gestational age, lower socioeconomic status and maternal education, longer total circulatory support time, a greater number of operations and catheterizations, longer hospital length of stay, and a greater number of complications.

### Outcomes Beyond Adolescence

As the population with Fontan circulation ages, further research is needed to assess the translation of neurodevelopmental and behavioral findings in childhood and adolescence into neurocognitive function in adulthood. Recent studies show that adults with CHD are at risk for persisting neurocognitive deficits, particularly in the domain of executive functions (69), which may alter their professional and personal achievement and quality of life. These deficits seem correlated to alterations of white matter microstructure in subjects with severe CHD, suggesting long-lasting neurological effects of critical CHD for some adults. A binational study using data from Australian and New Zealand Fontan Registry found that young adults with Fontan circulation obtained worse neurocognitive outcomes in several domains compared to those with d-transposition of the great arteries. Moreover, within group comparisons for the Fontan group found that adults with Fontan circulation had more severe impairments than Fontan adolescents in psychomotor function and working memory, a domain of executive functioning. Neurocognitive deficits were associated with reduced gray and white matter brain volumes in the Fontan group (26).

Finally, recognition and management of the neuropsychological and behavioral impairments that affect many but not all patients with Fontan circulation have important implications for patient education, medical adherence, the transition from pediatric to adult healthcare systems, completion of higher education, and adult employment. It also plays a critical role for the long-term mental health outcomes of this population.

## Mental Health in Patients With a Fontan Circulation

Living with a chronic health condition, including with Fontan circulation, elevates the risk of psychosocial difficulties in children, adolescents and adults. As a group, many children and adolescents with CHD are at risk of psychosocial adjustment difficulties, particularly according to parental reports (70). DeMaso and colleagues (64) compared the psychiatric and psychosocial status of 156 patients (mean age, 15 years) who had undergone Fontan procedures with that of 111 healthy peers. The patient cohort had a significantly higher rate of lifetime psychiatric diagnosis (65 vs. 22%); anxiety and attention-deficit/hyperactivity disorder were most common. Furthermore, as a group, patients had worse outcomes on measures of global psychosocial functioning, anxiety, depressive symptoms, post-traumatic stress, and disruptive behavior. Risk factors of poorer psychiatric/psychosocial outcomes include male sex, lower birth weight, longer duration of deep hypothermic circulatory arrest, and lower intelligence.

Conversely, in adults, a meta-analysis of international studies using survey assessment of psychological distress revealed no consistent evidence of poorer outcomes among adults with CHD of various subtypes, although methodological heterogeneity was evident (71). The results of 3 North American studies, however, suggest that one third of adults with CHD meet diagnostic criteria for an anxiety disorder when clinical interviews are administered (72–74). Indeed, depression may not be the most prevalent psychiatric disorder in the population with critical CHD. Adults with CHD are particularly at risk of elevated anxiety and post-traumatic stress disorder (74–76). Patients' subjective health status has been more strongly associated with psychosocial outcomes than objective assessment (77), which is an important consideration for physicians faced with the symptomatic adult patient with Fontan circulation. Among adolescents and young adults with Fontan physiology, elevated symptoms of depression have been reported (28 with mild symptoms, 32% with moderate symptoms) and demonstrated to be a negative predictor of quality of life (78). Furthermore, more than half of patients endorsed worry about current health, employment, and living independently (79). MRI of the brain in adolescents with a single ventricle reveals injury in select areas that control anxiety and depression, suggesting a structural basis for the functional deficits seen (80).

Improving the understanding and clinical treatment of neurological and mental health outcomes in adults with CHD is a research priority (81). Congenital cardiologist and related professionals working with people with a Fontan circulation are encouraged to be proactive by developing efficient ways to identify psychosocial adjustment issues, collaborating with psychologists and psychiatrists to prevent mental health morbidities.

## Conclusions

Patients with single ventricle are at high risk for neurologic, developmental, and psychosocial sequelae. A variety of developmental and injurious brain processes may occur, beginning during fetal development. Altered hemodynamics reduce cerebral substrate delivery, leading to abnormal brain growth and development as early as the second trimester of pregnancy. Specific forms of brain injury, such as white matter injury, stroke, or hemorrhage, may occur before, during, or after surgery. Importantly, in neonates with hypoplastic left heart syndrome, who have no medical contraindication, shorten time between birth and surgery may lower the risk of postoperative white matter injury, suggesting a potential neuroprotective effect (82).

Innate patient-related variables, including genetic factors, fetal growth, preterm delivery, and maternal education, may be the most important predictors of future neurodevelopmental outcome. Perioperative factors, such as multiple catheterizations and surgeries, as well as prolonged hospital stay, have been associated with adverse long-term neurodevelopmental outcome. Awareness and recognition of neuropsychological challenges in patients with Fontan circulation can facilitate counseling and increase access to diagnostic assessment and educational resources (4). Periodic surveillance, screening, and evaluation for neurodevelopmental disabilities starting in early life and beyond are recommended (3, 83, 84). Identification of deficits allows appropriate therapies and patient/community education and creates opportunities to enhance academic, behavioral, psychosocial, and adaptive functioning, thus allowing each individual to achieve his or her optimal potential. Finally, recognizing the remarkable resilience of patients with single ventricle disease throughout their lives serves as inspiration to strive for innovative strategies to better support their long-term quality of life.

## Author Contributions

JC and CR wrote the initial draft of the manuscript and revised it and accepted for publication. JN supervised and critically revised the manuscript and accepted for publication. All authors contributed to the article and approved the submitted version.

## Funding

This work was supported by The Farb Family Fund.

## Conflict of Interest

The authors declare that the research was conducted in the absence of any commercial or financial relationships that could be construed as a potential conflict of interest.

## Publisher's Note

All claims expressed in this article are solely those of the authors and do not necessarily represent those of their affiliated organizations, or those of the publisher, the editors and the reviewers. Any product that may be evaluated in this article, or claim that may be made by its manufacturer, is not guaranteed or endorsed by the publisher.

## References

[1] Rychik J Atz AM Celermajer DS Deal BJ Gatzoulis MA Gewillig MH. Evaluation and management of the child and adult with Fontan circulation: a scientific statement from the American heart association. Circulation. (2019) 140:e234–84. 10.1161/CIR.0000000000000696 31256636

[2] Zentner D Celermajer DS Gentles T d'Udekem Y Ayer J Blue GM. Management of people with a fontan circulation: a cardiac society of australia and new zealand position statement. Heart Lung Circ. (2020) 29:5–39. 10.1016/j.hlc.2019.09.010 31735685

[3] Marino BS Lipkin PH Newburger JW Peacock G Gerdes M Gaynor JW. American heart association congenital heart defects committee, council on cardiovascular disease in the young, council on cardiovascular nursing, council on cardiovascular disease in the young, council on cardiovascular nursing, and stroke council, neurodevelopmental outcomes in children with congenital heart disease: evaluation and management: a scientific statement from the american heart association. Circulation. (2012) 126:1143–72. 10.1161/CIR.0b013e318265ee8a 22851541

[4] Sood E Jacobs JP Marino BS. The cardiac neurodevelopmental outcome collaborative: a new community improving outcomes for individuals with congenital heart disease. Cardiol Young. (2020) 30:1595–6. 10.1017/S1047951120003509 33269668

[5] Newburger JW Sleeper LA Bellinger DC Goldberg CS Tabbutt S Lu M. Early developmental outcome in children with hypoplastic left heart syndrome and related anomalies: the single ventricle reconstruction trial. Circulation. (2012) 125:2081–91. 10.1161/CIRCULATIONAHA.111.064113 22456475 PMC3341507

[6] Marelli A Miller SP Marino BS Jefferson AL Newburger JW. Brain in congenital heart disease across the lifespan: the cumulative burden of injury. Circulation. (2016) 133:1951–62. 10.1161/CIRCULATIONAHA.115.019881 27185022 PMC5519142

[7] Homsy J Zaidi S Shen Y Ware JS Samocha KE Karczewski KJ. *De novo* mutations in congenital heart disease with neurodevelopmental and other congenital anomalies. Science. (2015) 350:1262–6. 10.1126/science.aac9396 26785492 PMC4890146

[8] Limperopoulos C Tworetzky W McElhinney DB Newburger JW Brown DW Robertson RL Jr. Brain volume and metabolism in fetuses with congenital heart disease: evaluation with quantitative magnetic resonance imaging and spectroscopy. Circulation. (2010) 121:26–33. 10.1161/CIRCULATIONAHA.109.865568 20026783 PMC2819908

[9] Bucholz EM Sleeper LA Newburger JW. Neighborhood socioeconomic status and outcomes following the Norwood procedure: an analysis of the pediatric heart network single ventricle reconstruction trial public data set. J Am Heart Assoc. (2018) 7:e007065. 10.1161/JAHA.117.007065 29420218 PMC5850235

[10] Calderon J Stopp C Wypij D DeMaso DR Rivkin M Newburger JW. Early-term birth in single-ventricle congenital heart disease after the Fontan procedure: neurodevelopmental and psychiatric outcomes. J Pediat. (2016) 179:96–103. 10.1016/j.jpeds.2016.08.084 27692462

[11] Goff DA Luan X Gerdes M Bernbaum J D'Agostino JA Rychik J. Younger gestational age is associated with worse neurodevelopmental outcomes after cardiac surgery in infancy. J Thorac Cardiovas Surg. (2012) 143:535–42. 10.1016/j.jtcvs.2011.11.029 22340027 PMC4423823

[12] Gaynor JW Wernovsky G Jarvik GP Bernbaum J Gerdes M Zackai E. Patient characteristics are important determinants of neurodevelopmental outcome at one year of age after neonatal and infant cardiac surgery. J Thorac Cardiovas Surg. (2007) 133:1344–53. 10.1016/j.jtcvs.2006.10.087 17467455 PMC2844117

[13] Mahle WT Lu M Ohye RG William Gaynor J Goldberg CS Sleeper LA. predictive model for neurodevelopmental outcome after the Norwood procedure. Pediatr Cardiol. (2013) 34:327–33. 10.1007/s00246-012-0450-1 22864647 PMC3505274

[14] Mahle WT Lu M Ohye RG William Gaynor J Goldberg CS Sleeper LA. predictive model for neurodevelopmental outcome after the Norwood procedure. Pediatric cardiology. (2013) 34:327–33. 10.1016/j.jpeds.2014.05.019 22864647 PMC3505274

[15] The The International Cardiac Collaborative on Neurodevelopment (ICCON) Investigators Impact Impact of operative and postoperative factors on neurodevelopmental outcomes after cardiac operations. Annals Thorac Surg. (201)6 102:843–9. 10.1016/j.athoracsur.2016.05.081 27496628

[16] Wernovsky G. Current insights regarding neurological and developmental abnormalities in children and young adults with complex congenital cardiac disease. Cardiol Young. (2006) 16:92–104. 10.1017/S1047951105002398 16401370

[17] Rollins CK Ortinau CM Stopp C Friedman KG Tworetzky W Gagoski B. Regional brain growth trajectories in fetuses with congenital heart disease. Ann Neurol. (2021) 89:143–57. 10.1002/ana.25940 33084086 PMC7970443

[18] Watson CG Stopp C Wypij D Newburger JW Rivkin MJ. Reduced cortical volume and thickness and their relationship to medical and operative features in post-Fontan children and adolescents. Pediatr Res. (2017) 81:881–90. 10.1038/pr.2017.30 28157834

[19] Licht DJ Shera DM Clancy RR Wernovsky G Montenegro LM Nicolson SC. Brain maturation is delayed in infants with complex congenital heart defects. J Thorac Cardiovas Surg. (2009) 137:529–37. 10.1016/j.jtcvs.2008.10.025 19258059 PMC2701902

[20] Miller SP McQuillen PS Hamrick S Xu D Glidden DV Charlton N. Abnormal brain development in newborns with congenital heart disease. New Eng J Med. (2007) 357:1928–38. 10.1056/NEJMoa067393 17989385

[21] Clouchoux C Du Plessis AJ Bouyssi-Kobar M Tworetzky W McElhinney DB Brown DW. Delayed cortical development in fetuses with complex congenital heart disease. Cerebral Cortex. (2013) 23:2932–43. 10.1093/cercor/bhs281 22977063

[22] Jaimes C Rofeberg V Stopp C Ortinau CM Gholipour A Friedman KG. Association of isolated congenital heart disease with fetal brain maturation. Am J Neuroradiol. (2020) 41:1525–31. 10.3174/ajnr.A6635 32646947 PMC7658885

[23] Sun L Macgowan CK Sled JG Yoo SJ Manlhiot C Porayette P. Reduced fetal cerebral oxygen consumption is associated with smaller brain size in fetuses with congenital heart disease. Circulation. (2015) 131:1313–23. 10.1161/CIRCULATIONAHA.114.013051 25762062 PMC4398654

[24] Ortinau CM Rollins CK Gholipour A Yun HJ Marshall M Gagoski B. Early-emerging sulcal patterns are atypical in fetuses with congenital heart disease. Cerebral cortex. (2019) 29:3605–16. 10.1093/cercor/bhy235 30272144 PMC6644862

[25] Watson CG Stopp C Wypij D Bellinger DC Newburger JW Rivkin MJ. Altered white matter microstructure correlates with IQ and processing speed in children and adolescents post-fontan. J Pediat. (2018) 200:140–9. 10.1016/j.jpeds.2018.04.022 29934026

[26] Watson CG Stopp C Wypij D Bellinger DC Newburger JW Rivkin MJ. Altered white matter microstructure correlates with IQ and processing speed in children and adolescents post-fontan. J Pediat. (2018) 200:140–9. 10.1161/CIRCULATIONAHA.120.048202 29934026

[27] Goff DA Shera DM Tang S Lavin NA Durning SM Nicolson SC. Risk factors for preoperative periventricular leukomalacia in term neonates with hypoplastic left heart syndrome are patient related. J Thorac Cardiovas Surg. (2014) 147:1312–8. 10.1016/j.jtcvs.2013.06.021 23879933 PMC3896504

[28] Block AJ McQuillen PS Chau V Glass H Poskitt KJ Barkovich AJ. Clinically silent preoperative brain injuries do not worsen with surgery in neonates with congenital heart disease. J Thorac Cardiovas Surg. (2010) 140:550–7. 10.1016/j.jtcvs.2010.03.035 20434174 PMC2917479

[29] Beca J Gunn JK Coleman L Hope A Reed PW Hunt RW. New white matter brain injury after infant heart surgery is associated with diagnostic group and the use of circulatory arrest. Circulation. (2013) 127:971–9. 10.1161/CIRCULATIONAHA.112.001089 23371931

[30] Dent CL Spaeth JP Jones BV Schwartz SM Glauser TA Hallinan B. Brain magnetic resonance imaging abnormalities after the Norwood procedure using regional cerebral perfusion. J Thorac Cardiovas Surg. (2006) 131:190–7 10.1016/j.jtcvs.2005.10.003 16399311

[31] Algra SO Jansen NJ van der Tweel I Schouten AN Groenendaal F Toet M. Neurological injury after neonatal cardiac surgery: a randomized, controlled trial of 2 perfusion techniques. Circulation. (2014) 129:224–33. 10.1161/CIRCULATIONAHA.113.003312 24141323

[32] Andropoulos DB Hunter JV Nelson DP Stayer SA Stark AR McKenzie ED. Brain immaturity is associated with brain injury before and after neonatal cardiac surgery with high-flow bypass and cerebral oxygenation monitoring. J Thorac Cardiovas Surg. (2010) 139:543–56. 10.1016/j.jtcvs.2009.08.022 19909994 PMC2827639

[33] Fenton KN Lessman K Glogowski K Fogg S Duncan KF. Cerebral oxygen saturation does not normalize until after stage 2 single ventricle palliation. Annals Thoracic Surg. (2007) 83:1431–6. 10.1016/j.athoracsur.2006.10.013 17383352

[34] Licht DJ Wang J Silvestre DW Nicolson SC Montenegro LM Wernovsky G. Preoperative cerebral blood flow is diminished in neonates with severe congenital heart defects. J Thorac Cardiovas Surg. (2004) 128:841–9. 10.1016/S0022-5223(04)01066-9 15573068

[35] Petit CJ Rome JJ Wernovsky G Mason SE Shera DM Nicolson SC. Preoperative brain injury in transposition of the great arteries is associated with oxygenation and time to surgery, not balloon atrial septostomy. Circulation. (2009) 119:709–16. 10.1161/CIRCULATIONAHA.107.760819 19171858 PMC2736632

[36] Galli KK Zimmerman RA Jarvik GP Wernovsky G Kuypers MK Clancy RR. Periventricular leukomalacia is common after neonatal cardiac surgery. J Thorac Cardiovas Surg. (2004) 127:692–704. 10.1016/j.jtcvs.2003.09.053 15001897

[37] Guo T Chau V Peyvandi S Latal B McQuillen PS Knirsch W. White matter injury in term neonates with congenital heart diseases: topology & comparison with preterm newborns. Neuroimage. (2019) 185:742–9. 10.1016/j.neuroimage.2018.06.004 29890324 PMC6289608

[38] Day RW Boyer RS Tait VF Ruttenberg HD Factors associated with stroke following the Fontan procedure. Pediat Cardiol. (1995) 16:270–5. 10.1007/BF00798060 8650012

[39] Barker PC Nowak C King K Mosca RS Bove EL Goldberg CS. Risk factors for cerebrovascular events following fontan palliation in patients with a functional single ventricle. Am J Cardiol. (2005) 96:587–91. 10.1016/j.amjcard.2005.04.025 16098317

[40] Mackay MT Wiznitzer M Benedict SL Lee KJ Deveber GA Ganesan V. International pediatric stroke study, arterial ischemic stroke risk factors: the international pediatric stroke study. Annals Neurol. (2011) 69:130–40. 10.1002/ana.22224 21280083

[41] Chen J Zimmerman RA Jarvik GP Nord AS Clancy RR Wernovsky G et al. Perioperative stroke in infants undergoing open heart operations for congenital heart disease. Ann Thorac Surg. (2009) 88:823–9. 10.1016/j.athoracsur.2009.03.030 19699905 PMC2840405

[42] Kaulitz R Ziemer G Rauch R Girisch M Bertram H Wessel A. Prophylaxis of thromboembolic complications after the Fontan operation (total *cavopulmonary anastomosis*). J Thorac Cardiovas Surg. (2005) 129:569–75. 10.1016/j.jtcvs.2004.08.045 15746740

[43] Mahle WT Tavani F Zimmerman RA Nicolson SC Galli KK Gaynor JW. An MRI study of neurological injury before and after congenital heart surgery. Circulation. (2002) 106:I-109-I-114. 10.1161/01.cir.0000032908.33237.b1 12354718

[44] McQuillen PS Barkovich AJ Hamrick SE Perez M Ward P Glidden DV. Temporal and anatomic risk profile of brain injury with neonatal repair of congenital heart defects. Stroke. (2007) 38:736–41. 10.1161/01.STR.0000247941.41234.90 17261728

[45] du Plessis AJ Chang AC Wessel DL Lock JE Wernovsky G Newburger JW. Cerebroavascular accidents following the Fontan operation. Pediatric Neurol. (1995) 12:230–6. 10.1016/0887-8994(95)00027-D 7619190

[46] Dowling MM Hynan LS Lo W Licht DJ McClure C Yager JY. International paediatric stroke study, international paediatric stroke study: stroke associated with cardiac disorders. Intern Sstroke: Official J Intern Stroke Soc. (2013) 8(Suppl A100):39–44. 10.1111/j.1747-4949.2012.00925.x 23231361 PMC4940849

[47] Bellinger DC Watson CG Rivkin MJ Robertson RL Roberts AE Stopp C Dunbar-Masterson. Neuropsychological status and structural brain imaging in adolescents with single ventricle who underwent the Fontan procedure. J Am Heart Assoc. (2015) 4:e002302. 10.1161/JAHA.115.002302 26667085 PMC4845289

[48] Hoffman JL Mack GK Minich LL Benedict SL Heywood M Stoddard GJ. Failure to impact prevalence of arterial ischemic stroke in pediatric cardiac patients over three decades. Cong Heart Dis. (2010) 6:211–8. 10.1111/j.1747-0803.2011.00510.x 21450034

[49] Rodan L McCrindle BW Manlhiot C MacGregor DL Askalan R Moharir M. Stroke recurrence in children with congenital heart disease. Annals Neurol. (2012) 72:103–11. 10.1002/ana.23574 22829272

[50] Chung MG Guilliams KP Wilson JL Beslow LA Dowling MM Friedman NR. Arterial ischemic stroke secondary to cardiac disease in neonates and children. Pediatric Neurol. (2019) 100:35–41. 10.1016/j.pediatrneurol.2019.06.008 31371125 PMC7034952

[51] Domi T Edgell DS McCrindle BW Williams WG Chan AK MacGregor DL. Frequency, predictors, and neurologic outcomes of vaso-occlusive strokes associated with cardiac surgery in children. Pediatrics. (2008) 122:1292–8. 10.1542/peds.2007-1459 19047248

[52] Khairy P Fernandes SM Mayer JE Jr Triedman JK Walsh EP Lock JE. Long-term survival, modes of death, and predictors of mortality in patients with Fontan surgery. Circulation. (2008) 117:85–92. 10.1161/CIRCULATIONAHA.107.738559 18071068

[53] Soul JS Robertson RL Wypij D Bellinger DC Visconti KJ du Plessis AJ. Newburger, Subtle hemorrhagic brain injury is associated with neurodevelopmental impairment in infants with repaired congenital heart disease. J Thorac Cardiovas Surg. (2009) 138:374–81. 10.1016/j.jtcvs.2009.02.027 19619781 PMC2752843

[54] Fogel MA Li C Elci OU Pawlowski T Schwab PJ Wilson F. Neurological injury and cerebral blood flow in single ventricles throughout staged surgical reconstruction. Circulation. (2017) 135:671–82. 10.1161/CIRCULATIONAHA.116.021724 28031423 PMC5309199

[55] Rogers BT Msall ME Buck GM Lyon NR Norris MK Roland JM. Neurodevelopmental outcome of infants with hypoplastic left heart syndrome. J Pediat. (1995) 126:496–8. 10.1016/S0022-3476(95)70478-7 7532710

[56] Goldberg CS Schwartz EM Brunberg JA Mosca RS Bove EL Schork MA. Neurodevelopmental outcome of patients after the fontan operation: a comparison between children with hypoplastic left heart syndrome and other functional single ventricle lesions. J Pediat. (2000) 137:646–52. 10.1067/mpd.2000.108952 11060530

[57] Sarajuuri A Jokinen E Mildh L Tujulin AM Mattila I Valanne L. Neurodevelopmental burden at age 5 years in patients with univentricular heart. Pediatrics. (2012) 130:e1636–46. 10.1542/peds.2012-0486 23166336

[58] Gaynor JW Ittenbach RF Gerdes M Bernbaum J Clancy RR McDonald-McGinn DM. Spray, Neurodevelopmental outcomes in preschool survivors of the Fontan procedure. J Thorac Cardiovas Surg. (2014) 147:1276–82; discussion 1282–3. 10.1016/j.jtcvs.2013.12.019 PMC5662937 24521968

[59] Ohye RG Gaynor JW Ghanayem NS Goldberg CS Laussen PC Frommelt PC. Design and rationale of a randomized trial comparing the Blalock-Taussig and right ventricle-pulmonary artery shunts in the Norwood procedure. J Thoracic Cardiovasc Surg. (2008) 136:968–75. 10.1016/j.jtcvs.2008.01.013 18954638 PMC2745283

[60] Calderon J Bonnet D Courtin C Concordet S Plumet MH Angeard N. Executive function and theory of mind in school-aged children after neonatal corrective cardiac surgery for transposition of the great arteries. Develop Med Child Neurol. (2010) 52:1139–44. 10.1111/j.1469-8749.2010.03735.x 20804511

[61] Calderon J Angeard N Moutier S Plumet MH Jambaque I Bonnet D Impact Impact of prenatal diagnosis on neurocognitive outcomes in children with transposition of the great arteries. J Pediat. (2012) 161:94–8. 10.1016/j.jpeds.2011.12.036 22284567

[62] Calderon J Willaime M Lelong N Bonnet D Houyel L Ballon M. Population-based study of cognitive outcomes in congenital heart defects. Arch Dis Child. (2018) 103:49–56. 10.1136/archdischild-2016-310830 28780508

[63] Cassidy AR White MT DeMaso DR Newburger JW Bellinger DC. Executive function in children and adolescents with critical cyanotic congenital heart disease. J Inter Neuropsychol Soc: JINS. (2015) 21:34–49. 10.1017/S1355617714001027 25487044 PMC4762262

[64] DeMaso DR Calderon J Taylor GA Holland JE Stopp C White MT. Psychiatric disorders in adolescents with single ventricle congenital heart disease. Pediatrics. (2017) 139:2241. 10.1542/peds.2016-2241 28148729 PMC5330395

[65] Bellinger DC. Are children with congenital cardiac malformations at increased risk of deficits in social cognition? Cardiol Young. (2008) 18:3–9. 10.1017/S104795110700176X 18093362

[66] Neal AE Stopp C Wypij D Bellinger AC Dunbar-Masterson C DeMaso DR. Predictors of health-related quality of life in adolescents with tetralogy of Fallot. J Pediat. (2015) 166:132–8. 10.1016/j.jpeds.2014.09.034 25444004 PMC4274240

[67] Moffitt T Arseneault E Belsky LD Dickson N Hancox RA Harrington H. A gradient of childhood self-control predicts health, wealth, and public safety. Proc Natl Acad Sci USA. (2011) 108:2693–8. 10.1073/pnas.1010076108 21262822 PMC3041102

[68] Calderon J Wypij D Rofeberg V Stopp C Roseman A Albers D. Bellinger, randomized controlled trial of working memory intervention in congenital heart disease. J Pediat. (2020) 227:191–8. 10.1016/j.jpeds.2020.08.038 32827526

[69] Ehrler M Schlosser L Brugger P Greutmann M Oxenius A Kottke R. Altered white matter microstructure is related to cognition in adults with congenital heart disease. Brain Commun. (2021) 3:fcaa224. 10.1093/braincomms/fcaa224 33501427 PMC7811757

[70] Latal B Helfricht S Fischer JE Bauersfeld U Landolt MA. Psychological adjustment and quality of life in children and adolescents following open-heart surgery for congenital heart disease: a systematic review. BMC Pediatr. (2009) 9:6. 10.1186/1471-2431-9-6 19161602 PMC2642822

[71] Downing TE Allen KY Goldberg DJ Rogers LS Ravishankar C Rychik J et al. Surgical and catheter-based reinterventions are common in long-term survivors of the fontan operation. Circ Cardiovasc Interv. (2017) 10:4942. 10.1161/CIRCINTERVENTIONS.116.004924 28851719

[72] Horner T Liberthson R Jellinek MS. Psychosocial profile of adults with complex congenital heart disease. Mayo Clinic Proceedings. (2000) 75:31–36. 10.4065/75.1.31 10630755

[73] Kovacs AH Saidi AS Kuhl EA Sears SF Silversides C Harrison JL et al. Depression and anxiety in adult congenital heart disease: predictors and prevalence. Int J Cardiol. (2009) 137:158–64. 10.1016/j.ijcard.2008.06.042 18707776

[74] Bromberg JI Beasley PJ D'Angelo EJ Landzberg M DeMaso DR Depression and anxiety in adults with congenital heart disease: a pilot study. Heart Lung. (2003) 32:105–10. 10.1067/mhl.2003.26 12734533

[75] Deng LX Khan AM Drajpuch D Fuller S Ludmir J Mascio CE et al. Prevalence and correlates of post-traumatic stress disorder in adults with congenital heart disease. Am J Cardiol. (2016) 117:853–7. 10.1016/j.amjcard.2015.11.065 26803381

[76] Eslami B. Correlates of posttraumatic stress disorder in adults with congenital heart disease. Congenit Heart Dis. (2017) 12:357–63. 10.1111/chd.12452 28217850

[77] Callus E Utens EM Quadri E Ricci C Carminati M Giamberti A et al. The impact of actual and perceived disease severity on pre-operative psychological well-being and illness behaviour in adult congenital heart disease patients. Cardiol Young. (2014) 24:275–82. 10.1017/S1047951113000218 23534397

[78] Pike NA Evangelista LS Doering LV Eastwood JA Lewis AB Child JS. Quality of life, health status, and depression: comparison between adolescents and adults after the Fontan procedure with healthy counterparts. J Cardiovasc Nurs. (2012) 27:539–46. 10.1097/JCN.0b013e31822ce5f6 21912272 PMC3460064

[79] Pike NA Evangelista LS Doering LV Koniak-Griffin D Lewis AB Child JS. Clinical profile of the adolescent/adult Fontan survivor. Cong Heart Dis. (2011) 6:9–17. 10.1111/j.1747-0803.2010.00475.x 21269408 PMC3177559

[80] Pike NA Roy B Gupta R Singh S Woo MA Halnon NJ et al. Brain abnormalities in cognition, anxiety, and depression regulatory regions in adolescents with single ventricle heart disease. J Neurosci Res. (2018) 96:1104–18. 10.1002/jnr.24215 29315714 PMC6103299

[81] Gurvitz M Burns KM Brindis R Broberg CS Daniels CJ Fuller SM et al. Emerging research directions in adult congenital heart disease: a report from an NHLBI/ACHA working group. Am J College Cardiol. (2016) 67:1956–64. 10.1016/j.jacc.2016.01.062 27102511 PMC4846980

[82] Lynch JM Buckley EM Schwab PJ McCarthy AL Winters ME Busch DR et al. Time to surgery and preoperative cerebral hemodynamics predict postoperative white matter injury in neonates with hypoplastic left heart syndrome. J Thorac Cardiovasc Surg. (2014) 148:2181–8. 10.1016/j.jtcvs.2014.05.081 25109755 PMC4254035

[83] Ware J Butcher JL Latal B Sadhwani A Rollins CK Soto CL et al. Neurodevelopmental evaluation strategies for children with congenital heart disease aged birth through 5 years: recommendations from the cardiac neurodevelopmental outcome collaborative. Cardiol Young. (2020) 30:1609–1622. 10.1017/S1047951120003534 33143781

[84] Ilardi D Sanz JH Cassidy AR Sananes R Rollins CK Shade CU et al. Neurodevelopmental evaluation for school-age children with congenital heart disease: recommendations from the cardiac neurodevelopmental outcome collaborative. Cardiol Young. (2020) 30:1623–36. 10.1017/S1047951120003546 33143766

